# The histone methyltransferase KMT2D maintains cellular glucocorticoid responsiveness by shielding the glucocorticoid receptor from degradation

**DOI:** 10.1016/j.jbc.2024.107581

**Published:** 2024-07-25

**Authors:** Chuan-Jin Wu, Ferenc Livak, Jonathan D. Ashwell

**Affiliations:** 1 Laboratory of Immune Cell Biology, Center for Cancer Research, National Cancer Institute, National Institutes of Health, Bethesda, Maryland, USA; 2 Laboratory of Genome Integrity Flow Cytometry Core, Center for Cancer Research, National Cancer Institute, National Institutes of Health, Bethesda, Maryland, USA

**Keywords:** glucocorticoids, apoptosis, glucocorticoid receptor, KMT2D, protein stability, lymphocytes, glucocorticoid resistance

## Abstract

Because of their ability to induce lymphocyte apoptosis, glucocorticoids (GC) are widely used to treat hematological malignancies such as lymphomas and multiple myeloma. Their effectiveness is often limited, however, due to the development of glucocorticoid resistance by a variety of molecular mechanisms. Here we performed an unbiased genome-wide CRISPR screen with the human T-cell leukemia cell line Jurkat to find previously unidentified genes required for GC-induced apoptosis. One such gene was *KMT2D* (also known as *MLL2* or *MLL4*), which encodes a histone lysine methyltransferase whose mutations are associated with a variety of cancers, blood malignancies in particular, and are considered markers of poor prognosis. Knockout of KMT2D by CRISPR/Cas9 gene editing in Jurkat and several multiple myeloma cell lines downregulated GR protein expression. Surprisingly, this was not due to a reduction in GR transcripts, but rather to a decrease in the protein's half-life, primarily due to proteasomal degradation. Reconstitution of KMT2D expression restored GR levels. In contrast to the known ability of KMT2D to control gene transcription through covalent histone methylation, KMT2D-mediated upregulation of GR levels did not require its methyltransferase activity. Co-immunoprecipitation and proximity ligation assays found constitutive binding of KMT2D to the GR, which was enhanced in the presence of GC. These observations reveal KMT2D to be essential for the stabilization of cellular GR levels, and suggest a possible mechanism by which KMT2D mutations may lead to GC resistance in some malignancies.

Glucocorticoids (GCs) are steroid hormones primarily produced by the adrenals under the control of the hypothalamic-pituitary-adrenal axis. GCs are secreted rhythmically in a circadian manner, and their levels increase dramatically in response to physical and psychological stressors (1). GCs function by binding and activating the glucocorticoid receptor (GR), a ligand-dependent transcription factor belonging to the nuclear receptor family (2, 3). The GR is encoded by the gene *NR3C1* and bears the characteristic nuclear receptor structure, an N-terminal transactivation domain, a central DNA binding domain, and a C-terminal ligand binding domain. In the absence of ligand, the GR is predominantly localized in the cytoplasm in complexes with chaperones such as heat-shock protein (HSP) 90, HSP70, p23, and immunophilins (FKBP51, FKBP52, and Cyp44). Upon ligand engagement, the GR undergoes conformational changes, disassociates from the multimeric complex, and translocates into the nucleus where it interacts with co-regulators and other transcription factors to activate or repress transcription of numerous genes in a coordinated fashion (4). Transcriptional activation is driven by the binding of GR dimers or tetramers to GC response elements (GRE), which causes conformational changes in the GR transactivation domain that allow the recruitment of co-activators and/or other transcription factors. In addition, the GR binds to negative GREs to inhibit gene transcription (5, 6).

GCs exert pleiotropic physiologic actions and have especially prominent effects on metabolism and immunity. Their most potent effect on immune function, and T cells in particular, is immunosuppression (5). Ligand-bound GR suppresses antigen-stimulated inflammation mediated by macrophages, dendritic cells, and epithelial cells, and weakens cytotoxic immune responses by diminishing interferon-γ production and inhibiting the development of CD4^+^ helper T cells, CD8^+^ cytotoxic T cells, and natural killer cells (2). GCs also induce apoptosis of lymphoid cells, a phenomenon of considerable biological and clinical significance (7). Biologically, GC-induced apoptosis has been implicated in the generation of the antigen-specific T-cell repertoire and regulation of immune responses (5, 7). Clinically, because of their lympholytic action, GCs are exploited to treat lymphoid malignancies. Synthetic GCs such as dexamethasone (Dex) and prednisolone, combined with other chemotherapeutic and immunosuppressing agents, are cornerstones in the treatment of multiple myeloma, acute lymphoblastic leukemia (ALL), and chronic lymphocytic leukemia (CLL) (8, 9). GC-based therapies, however, may be hindered by GC-related side effects and GC resistance. GC resistance may be congenital or acquired during prolonged GC treatment (10). Deciphering the mechanisms of GC-induced apoptosis could yield valuable information for the understanding of GC resistance.

Despite much effort, the mechanisms governing GC-induced apoptosis are far from fully understood. GR-mediated gene activation is essential for the initiation of the process, as GC-induced apoptosis is inhibited by actinomycin D and cycloheximide, which block gene transcription and protein synthesis, respectively (8). It is generally agreed that GC-induced apoptosis primarily involves the intrinsic mitochondrial apoptotic pathway (8). Although no GRE is found in the promoter region, several studies reported that the pro-apoptotic Bcl2 family member *BCL2L11* (BIM) was upregulated by GCs and thus implicated in GC-induced apoptosis (11, 12, 13). Several GR target genes such as *GILZ* (14), *SARI* (15), *TXNIP* (16), *ZBTB16* (17), and *Noxa* (18) have also been linked to GC-mediated cell killing. The proposed mechanisms of GC resistance include reduced GR expression, decreased GR affinity, impaired GR translocation, altered GR post-translational modifications (PTMs), altered GR transcriptional activity, altered expression of GR isoforms, and more broadly epigenetic changes, signaling pathway interactions, and deregulated metabolism (8, 19).

In an attempt to find new genes that regulate GC-induced apoptosis, we performed a genome-wide CRISPR screen on Jurkat T-lymphoid leukemia cells. We identified the histone methyltransferase *KMT2D* to be essential for GC-induced cell death in multiple lymphoma and multiple myeloma cells. Mechanistically, KMT2D acts at the post-translational level by protecting the GR from ubiquitination and proteasomal degradation. These findings uncover a novel GR regulatory mechanism and provide new insight into the acquisition of GC resistance.

## Results

### CRISPR-Cas9-based screen for GC-responsive pro-apoptotic genes

**Figure 1:**
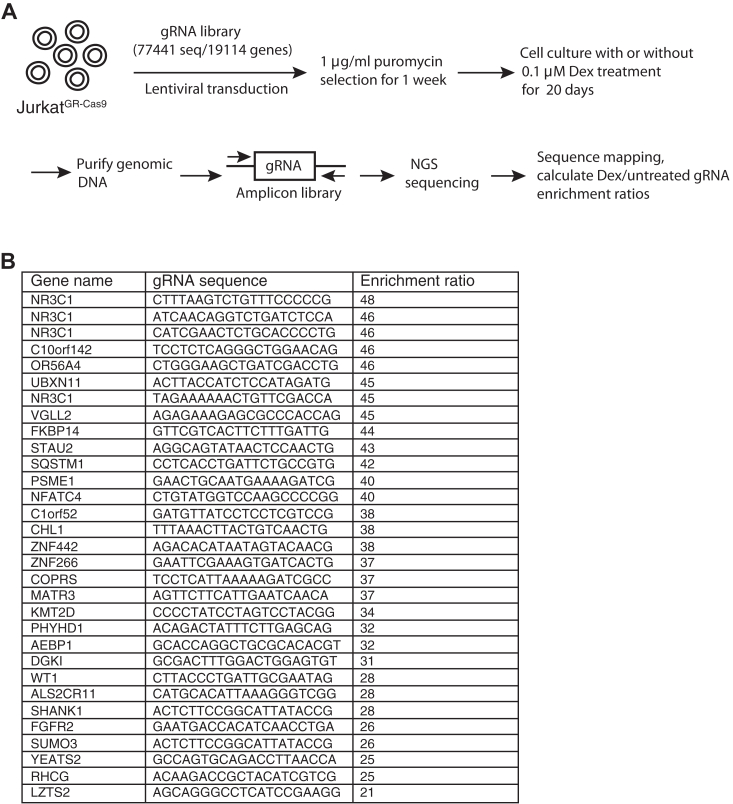
**CRISPR screen to identify genes involved in GC-induced apoptosis.***A*, schematic representation of the CRISPR screen strategy. Jurkat^GR-Cas9^ cells were transduced with the Brunello lentiviral gRNA library and selected with puromycin. The bulk gRNA sequences extracted from the genome DNA of cells grown in medium and those that survived for 20 days in the presence of 100 nM Dex were read by high-throughput next-generation sequencing. *B*, the names and gRNA targeting sequences of gRNAs that were enriched >20-fold in the Dex-treated group compared to control.

To identify genes required for GC-induced apoptosis, we performed a genome-wide CRISPR screen using the human T leukemia cell line Jurkat (Fig. 1*A*). Because native Jurkat cells express low levels of GR that limit their responses to GC (20), we transduced them with a human GR expression vector and selected clones, termed Jurkat^GR^, that uniformly express the GR at high levels (Fig. S1*A*). The use of cells in which GR transcription is controlled by a heterologous rather than native promoter has the theoretical advantage that genes involved in endogenous GR transcription would likely not be identified in the CRISPR screen. Jurkat^GR^ cells were transduced with a Cas9 expression vector to obtain a stable Jurkat^GR-Cas9^ clone to be used for screening. Whereas Jurkat^WT^ cells were resistant to even micromolar amounts of dexamethasone (Dex), Jurkat^GR^ and Jurkat^GR-Cas9^ cells were efficiently killed by nanomolar concentrations (Fig. S1, *B* and *C*), which is consistent with the observation by Helmberg *et al.* (20). Jurkat^GR-Cas9^ cells were transduced with a Brunello pooled sgRNA CRISPR library that targets nearly every human protein-coding gene with 4 different single guide (sg) RNAs. After 1-week puromycin selection for gRNA-expressing cells, there was a secondary selection with 100 nM Dex for 20 days to eliminate any glucocorticoid-sensitive cells. Sequencing of CRISPR sgRNAs identified 28 unique genes that were enriched >20-fold in Dex-treated compared to untreated cells (Fig. 1*B*). Three of the four most enriched sgRNA sequences targeted *NR3C1*, which encodes the GR, validating the selective pressure of glucocorticoid-mediated cell killing. Interestingly, despite being known to be involved in GC-induced apoptosis, very few sgRNAs that target genes encoding BCL-2 family members or caspases had enrichment ratios >3 (Table S1). This may suggest that at least using these selecting strategies, eliminating a single apoptosis-mediating or executing molecule is not sufficient to prevent GC-induced cell death.

### KMT2D sensitizes cells to GC-induced apoptosis

**Figure 2:**
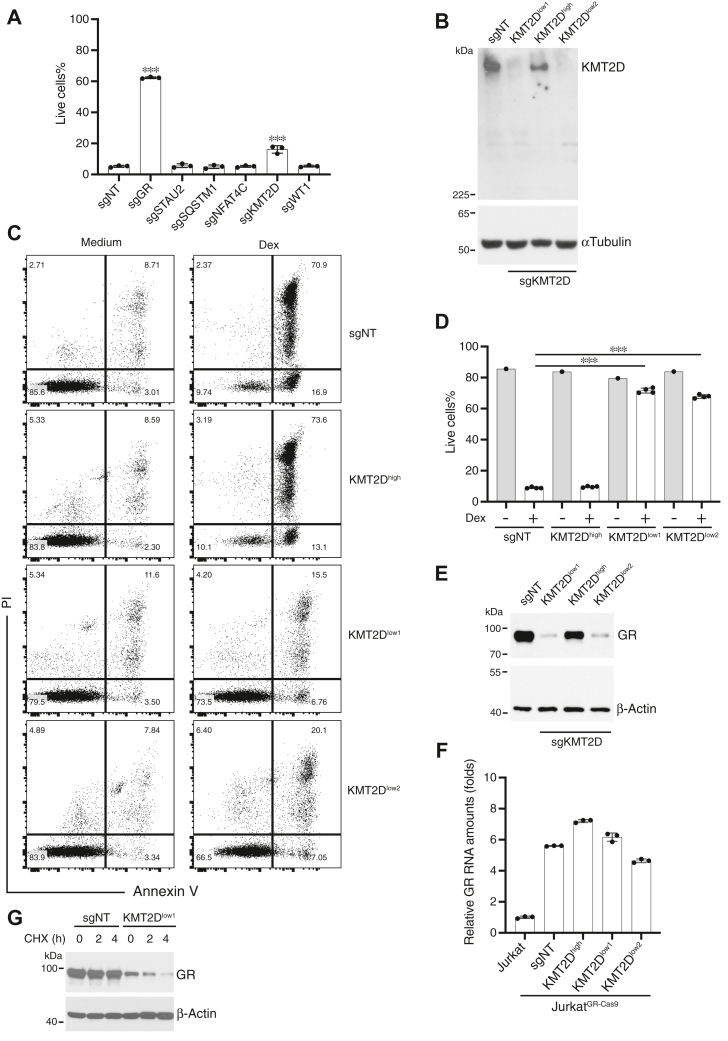
**Cells lacking KMT2D express much lower levels of GR and are insensitive to GC-induced cell death**. *A*, verification of a role for candidate sgRNAs in GC-induced apoptosis. CRISPR vectors containing oligonucleotides of individual candidate sgRNAs, one of the 4 *NR3C1*-targeting sgRNAs from the Brunello gRNA library (serving as a positive control), and a non-targeting sgRNA sequence (sgNT) were individually transduced into Jurkat^GR-Cas9^ cells. Cells that survived puromycin selection were treated with or without 100 nM Dex for 40 h and stained with PI and Annexin V-FITC. Cells negative for both were considered live. Live cell percentages (mean ± SD, data from 3 independent experiments) are depicted. ∗∗∗*p* < 0.001. *B*, cloned KMT2D Jurkat^GR^ cells. *KMT2D* sgRNA-transduced Jurkat^GR-Cas9^ cells were cloned by limiting dilution, and KMT2D levels in the indicated subclones were quantitated by immunoblotting. *C* and *D*, the cells in *panel B* were cultured in medium alone or with 20 nM Dex for 40 h and stained with PI and Annexin V-FITC. Representative plots are shown in (*C*), and the percentages of live cells (mean ± SD, n = 4) are shown in (*D*). ∗∗∗*p* < 0.001. *E*, GR levels were quantitated in the indicated cells by immunoblotting. One representative experiment of 3 is shown. *F*, *NR3C1* levels (mean ± SD, n = 3) in the indicated cells were measured by qRT-PCR. *G*, cells were incubated with 100 μg/ml cycloheximide (CHX) for the indicated times and immunoblotted for GR.

Reasoning that novel regulators of GC-induced apoptosis would be more likely to be found among genes whose products affect gene transcription or cell viability, CRISPR screen-identified sgRNAs that target tumor suppressors (KMT2D and WT1), the transcription factor (NFAT4C), an autophagy-regulating protein (p62/SQSTM1), and an RNA-binding protein (STAU2) were tested by delivering them individually into Jurkat^GR-Cas9^ cells. Serving as a positive control, pooled cells expressing sgRNA targeting *NR3C1* were resistant to Dex-induced apoptosis. Of the gene-specific sgRNAs tested, only pools of Jurkat^GR-Cas9^ cells transduced with sg*KMT2D* were statistically significantly resistant to Dex-induced apoptosis compared to non-targeting sgRNA-transduced cells (Fig. 2*A*). The sg*KMT2D* transfectants were subcloned by limiting dilution and immunoblotted for expression of KMT2D. Three subclones were chosen for study, one that expressed KMT2D (KMT2D^high^) and two in which KMT2D was undetectable (KMT2D^low^) (Fig. 2*B*). After 40 h of culture in medium or with 20 nM Dex, the cells were stained with Annexin V-FITC and propidium iodide and analyzed by flow cytometry (Fig. 2, *C* and *D*). Dex induced the death of > 60% of KMT2D-expressing cells (sgNT-transduced Jurkat^GR-Cas9^ cells and KMT2D^high^) but <15% of cells in which KMT2D expression was undetectable, indicating that KMT2D is a positive regulator of GC-induced apoptosis.

### KMT2D maintains GR protein levels in Jurkat T-leukemia cells

KMT2D (also known as MLL2 or MLL4) belongs to the COMPASS (COMplex of Proteins ASsociated with Set1) class of lysine methyltransferases (KMT) family, which carries out the methylation of histone H3 lysine 4 (H3K4). The KMT2C/D subfamily catalyzes the monomethylation of H3K4 and regulates gene expression through epigenetic modification of chromatin around enhancer regions (21). KMT2D is considered a tumor suppressor gene, and its mutations are associated with non-Hodgkin lymphoma, including diffuse large B-cell (DLBCL) and follicular lymphoma, as well as some solid tumors such as small cell lung cancer (22, 23). It has been reported that KMT2D-mediated epigenetic changes assist in the transcription of estrogen receptor (ER)-dependent genes (24). Based on these findings, it appeared likely that the requirement for KMT2D in GC-induced death may be due to its effect on GR-transcriptional activity as well. Surprisingly, however, we found that the loss of KMT2D led to reduced expression of the GR protein itself (Fig. 2*E*). Because GR expression in the Jurkat^GR^ cells is predominantly driven by the exogenous viral LTR5 promoter (Fig. S1*A*), it seemed unlikely that the decrease in GR protein caused by *KMT2D* deletion was due to the downregulation of transcription. Indeed, qRT-PCR analysis found that *NR3C1* mRNA levels were relatively similar between KMT2D-positive and KMT2D-deficient cells (Fig. 2*F*). To assess a post-transcriptional role for KMT2D, the kinetics of GR protein turnover were determined by assessing its levels over time after blocking new protein synthesis with cycloheximide. The GR level in KMT2D^low^ Jurkat^GR^ cells was low, as expected, and was almost completely undetectable after 4 h (Fig. 2*G*). GR expression in KMT2D-sufficient control cells, on the other hand, was stable over this time course. These results indicate that KMT2D does not affect *NR3C1* transcription but rather the stability of the GR protein.

**Figure 3:**
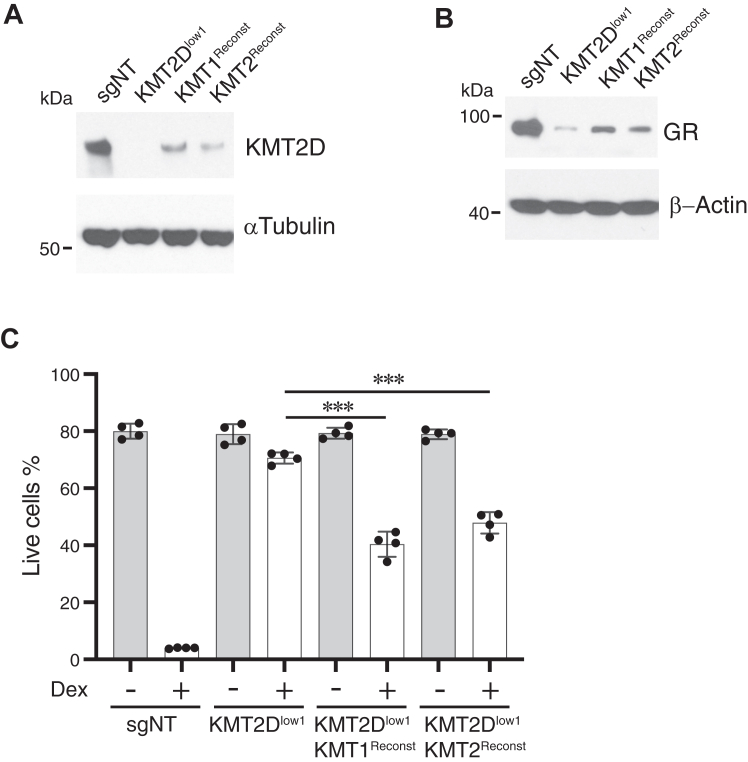
.**KMT2D-reconstitution in KMT2D-deficient cells restores GR expression and GC sensitivity**. KMT2D^Low1^ Jurkat^GR^ cells were transfected with a mammalian expression vector containing *KMT2D* in which the sgRNA targeting and PAM sequence were mutated to avoid deletion of the transfected gene, selected with puromycin, and cloned by limiting dilution. Thirty-five individual clones were screened for KMT2D expression by immunoblotting, and the two having the highest KMT2D levels, KMT1^Reconst^ and KMT2^Reconst^, were chosen for further analysis. *A* and *B*, immunoblots for (*A*) KMT2D and (*B*) GR. One representative experiment of 3 is shown. *C*, survival (mean ± SD, n = 4) of the indicated clones after 40 h of culture in medium alone or in the presence of 50 nM Dex. ∗∗∗*p* < 0.001.

To address the possibility that the phenotype of cells in which KMT2D was targeted was due to sgRNA off-target effects, KMT2D-deficient Jurkat cells were reconstituted by transfection with codon-optimized *KMT2D* expression plasmid. We were able to identify and isolate two (of 35 screened) independent KMT2D-expressing subclones for analysis. Despite KMT2D levels being much lower than in not-targeted cells (Fig. 3*A*), likely due to the size of its coding sequence (16,614 nucleotides), GR expression was partially restored to levels sufficient to render them sensitive to Dex-induced apoptosis, although less so than control cells (Fig. 3, *B* and *C*). Together, the results demonstrate that KMT2D supports GR-mediated apoptosis, at least in part, by increasing GR protein levels.

### Overexpression of KMT2D upregulates GR expression

**Figure 4:**
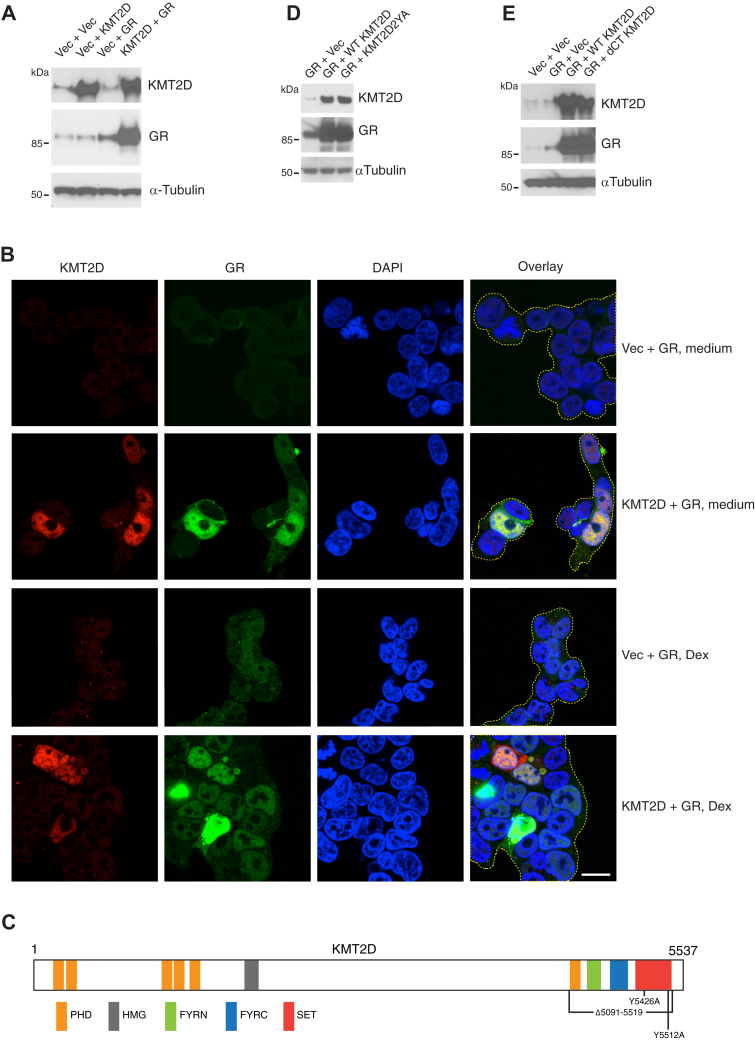
**KMT2D methyltransferase activity is not required for the effect of KMT2D on GR expression.** 293T cells were transfected with mammalian expression plasmids containing the indicated genes. *A*, cell lysates obtained 48 h after transfection were immunoblotted for KMT2D or GR. *B*, 293T cells transfected as indicated were cultured for 72 h at 37 °C and then exposed or not to 100 nM Dex for 30 min. Cells were fixed, permeabilized, stained with antibodies to KMT2D and GR and fluorescently labeled secondary anti-mouse and anti-rabbit antibodies, and visualized by confocal microscopy. Cell boundaries were depicted with *dotted yellow lines*. Scale bar, 20 μm. *C*, schematic representation of KMT2D protein domain organization and mutations. *D* and *E*, 293T cells were co-transfected with a GR expression vector and plasmids containing WT *KMT2D* or the methyltransferase-deficient mutant *KMT2D*^Y5426A,Y5512A^ (*D*), or in which most of the C-terminal-encoding sequence was deleted, *KMT2D*^*Δ5091-5519*^ (*E*). Cell lysates were immunoblotted for KMT2D and GR. Tubulin was used as a loading control. One representative experiment of 2 or 3 is shown for each panel.

To ask if this unexpected relationship between KMT2D and GR occurs in other cells, we took a reciprocal approach, overexpressing rather than targeting KTM2D, in human embryonic kidney (HEK) 293T cells. Unmanipulated 293T cells expressed little KMT2D or GR, and as assessed by immunoblotting there was little change in the latter when KMT2D was introduced (Fig. 4*A*). Transfection with the GR-expression plasmid alone had a modest effect, but in combination with KMT2D, there was a very large increase in GR levels. The synergy between GR and KMT2D was also observed in individual cells using confocal microscopy (Figs. 4*B* and S2). As expected, the majority of KMT2D localized in the nucleus, whereas GR expressed alone localized to the cytoplasm. Unexpectedly, the GR colocalized with KMT2D in the nucleus in most GR and KMT2D co-expressing cells even in the absence of Dex (Figs. 4*B* and S2).

### Methyltransferase activity is not required for KMT2D-dependent regulation of GR levels

To determine if KMT2D enzymatic activity plays a role in the stabilization of GR, WT, or a methyltransferase-inactive KMT2D mutant containing two amino acid substitutions in the catalytic domain (KMT2D^Y5426A/Y5512A^) (25) (Fig. 4*C*) were co-expressed with GR in 293T cells (Fig. 4*D*). Both WT and mutant proteins were expressed at similar levels and resulted in a comparable increase in co-transfected GR, indicating that the effect of KMT2D is independent of its histone-modifying enzymatic activity. Many of the predicted structural features of KMT2D, including the SET domain that contains the catalytic site, are localized near the C-terminus (23) (Fig. 4*C*). We created a construct in which most of the KMT2D C-terminus (residues 5091–5519) was deleted including the C-terminal PHD, FY-rich, and SET domains (Fig. 4*D*). This protein still retained the capacity to upregulate GR protein levels to the same extent as the full-length KMT2D (Fig. 4*E*).

### Knockout of KMT2D reduces endogenous GR protein expression in myelomas and B-cell lymphomas

**Figure 5:**
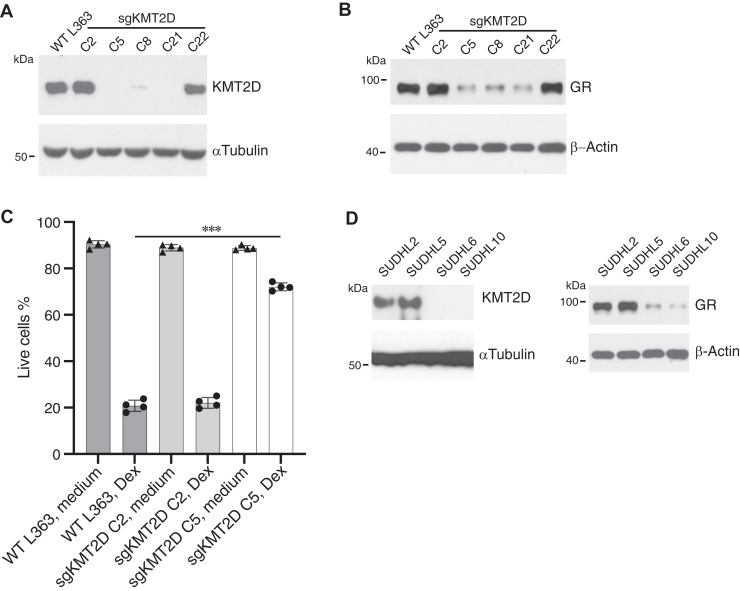
**KMT2D regulation of endogenous GR**. *A*, CRISPR/Cas9 was used to target *KMT2D* in L363 multiple myeloma cells. Subclones were analyzed for KMT2D (*A*) and GR (*B*) by immunoblotting. *C*, the indicated L363 variants were cultured with or without 100 nM Dex for 40 h and stained with PI/Annexin-V-FITC. The fraction of live cells (mean ± SD, n = 4) is shown. ∗∗∗*p* < 0.001. *D*, SUDHL DLBCL cells were immunoblotted for KMT2D and GR. The data are representative of 3 independent experiments.

Synthetic GCs are an integral component in the treatment of multiple myeloma, and low GR levels in tumor cells are associated with inferior survival outcomes (9). To assess if endogenous KMT2D regulates the expression of endogenous GR in these cells, we used CRISPR/Cas9 to target *KMT2D* in L363 cells, a GC-sensitive multiple myeloma cell line (26). Knockout of KMT2D in three independent subclones (C5, C8, and C21) corresponded with a marked decrease in GR expression compared to parental L363 cells and the two subclones (C2 and C22) in which KMT2D levels were not affected (Fig. 5, *A* and *B*). Cellular GR levels corresponded well with sensitivity to Dex-induced apoptosis (Fig. 5*C*). KMT2D protein levels are reportedly to diminished or absent in many diffuse large B cell lymphoma (DLBCL) cell lines due to *KMT2D* mutations (27). We investigated the relationship between KMT2D and GR levels in four DLBCL lines. KMT2D was easily detectable in SUDHL2 and SUDHL5 but absent in SUDHL6 and SUDHL10 cells (Fig. 5*D*). In agreement with the myeloma cells, expression of endogenous GR was high in the KMT2D^+^ DLBCL lines and much lower in the KMT2D^-^ DLBCL lines. Taken together, the results indicated that GR protein levels are maintained by endogenously expressed KMT2D in multiple different lymphoid malignancies.

### KMT2D protects the GR from proteasomal degradation

**Figure 6:**
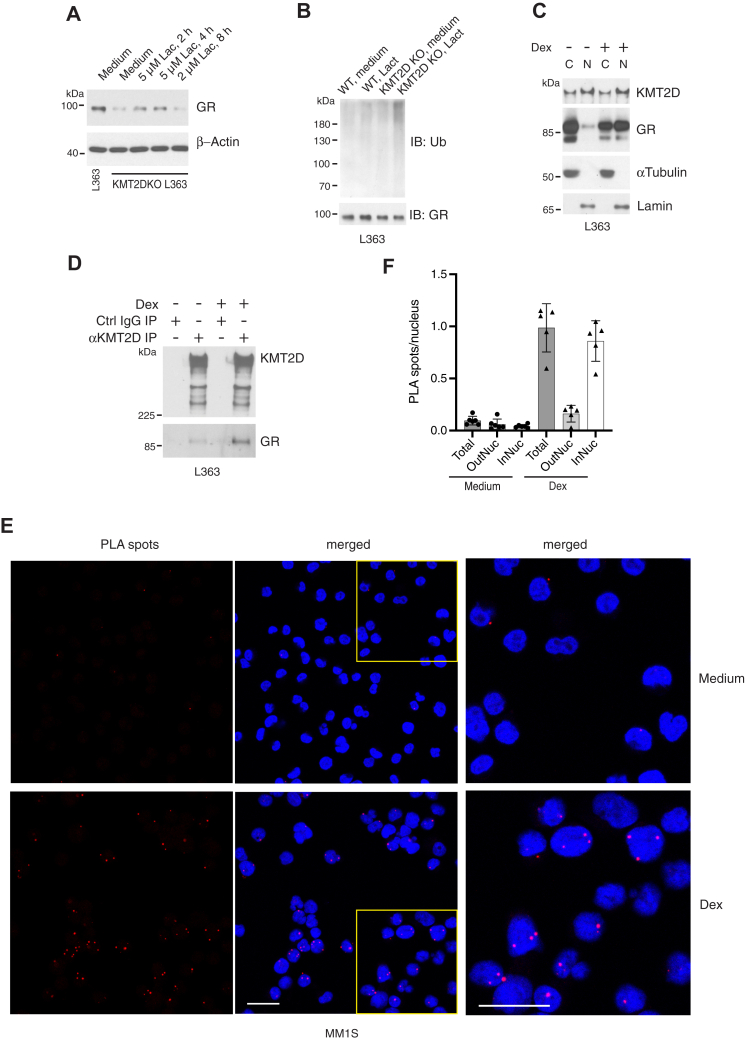
**KMT2D interacts with and protects the GR from proteasomal degradation.***A*, WT and KMT2D-deficient L363 cells were cultured with lactacystin (Lac) at the indicated concentrations and durations, and then immunoblotted for GR. *B*, WT and KMT2D-deficient L363 were incubated with 5 μM lactacystin for 3 h, at which time the GR was immunoprecipitated from RIPA cell lysates. The lysates were brought to an SDS concentration of 1% and heated at 95 °C for 5 min to dissociate co-immunoprecipitated proteins, then diluted with buffer to 0.1% SDS, immunoprecipitated with anti-GR antibody, and immunoblotted for ubiquitin and GR. *C*, L363 cells treated with or without 100 nM Dex for 30 min were fractionated into cytosolic and nuclear compartments and immunoblotted for KMT2D and GR. *D*, L363 cells were cultured with or without 100 nM Dex for 30 min. Anti-KMT2D or control IgG immunoprecipitates were resolved by SDS-PAGE and immunoblotted for KMT2D and GR. *E*, MM1S multiple myeloma cells cultured in chamber slides were cultured with 100 nM Dex at 37 °C for 30 min. The cells were fixed, stained, and PLA spots were visualized by confocal fluorescence microscopy. Higher magnification versions of outlined regions in the middle column are shown on the *right*. The images are representative of 2 independent experiments. Scale bars, 20 μm. *F*, PLA spots were analyzed and quantitated using Imaris software. The numbers (mean ± SD, n = 5) of the number of PLA spots outside (OutNuc) or inside (InNuc) nuclei per nucleus is depicted.

It has been reported that the GR is ubiquitinated and degraded in proteasomes following GC stimulation (28, 29). Ubiquitination of the GR has also been detected in cells under normal culture conditions, and proteasomal inhibition resulted in increased steady-state GR levels (30). To ask how KMT2D prolongs GR half-life (Fig. 2*G*), KMT2D-deficient Jurkat^GR^ and L363 cells were cultured with inhibitors of proteasomes (MG-132 or lactacystin), lysosomes (chloroquine), or autophagy (bafilomycin A). Proteasome inhibition was able to partially rescue GR levels in KMT2D^low1^ and KMT2D-deficient L363 cells (Figs. S3*A* and 6*A*). Culturing these cells for longer periods with lactacystin or MG-132 was toxic, perhaps due to proteotoxic stress and an excessive unfolded protein response (31). In contrast, neither bafilomycin A nor chloroquine affected GR loss (Fig. S3*B*). GR ubiquitination was assessed by immunoblotting immunoprecipitated GR with anti-ubiquitin antibodies. To eliminate the possibility that the ubiquitination bands may come from co-immunoprecipitated proteins, the cell lysates were heated to 95 °C in RIPA buffer containing 1% SDS to disrupt protein complexes prior to immunoprecipitation. The basal level of GR ubiquitination was unaffected by proteasomal inhibition in L363 cells, suggesting a low rate of steady-state ubiquitination (Fig. 6*B*). In KMT2D-deficient L363 cells, on the other hand, inhibition of proteasomal degradation resulted in a clear increase in the detection of ubiquitinated GR species, consistent with the notion that KMT2D protects the GR from ubiquitination-dependent turnover.

KMT2D is a histone methyltransferase that is active on chromatin in the nucleus, whereas in the absence of ligand, the GR is primarily cytosolic. Indeed, when cytosolic and nuclear fractions from L363 cells were compared more KMT2D was found in the nucleus, but a substantial amount was also detected in the cytosolic fraction (Fig. 6*C*). As expected, the GR was predominantly cytosolic in unstimulated cells, with a small amount present in the nuclear fraction (Fig. 6*C*). Similar results were obtained with Jurkat^GR^ cells (Fig. S3*C*). Because ectopic expression of proteins can lead to aberrant trafficking and intracellular localization, we asked if we could detect interactions of endogenous proteins by co-immunoprecipitation (IP). To extract proteins from the nucleus as well as cytoplasm, L363 cells were lysed with RIPA buffer. Immunoblotting of anti-KMT2D IP detected a faint co-immunoprecipitated GR band, which was enhanced when cells were cultured with Dex (Fig. 6*D*). Considering that RIPA lysis buffer contains 0.1% SDS which tends to disrupt noncovalent protein-protein interactions, we also carried out proximity ligation assays (PLA), which detect protein interactions *in situ* (32). For these studies another GC-sensitive multiple myeloma cell line, MM1S, was chosen because unlike L363 it is adherent and easier to observe using fluorescence microscopy. MM1S cells expressed easily detectible GR and KMT2D (Fig. S3*D*) and are sensitive to GC-induced apoptosis (26). Few if any PLA signals were detected when single antibodies were used (Fig. S3*E*). When PLA was performed with anti-KMT2D and anti-GR, a few PLA spots were detected in unstimulated cells, primarily at the nuclear periphery and/or cytoplasm (Fig. 6*E*). The number of PLA spots markedly increased within the nucleus by stimulation with Dex (Fig. 6, *E* and *F*).

**Figure 7:**
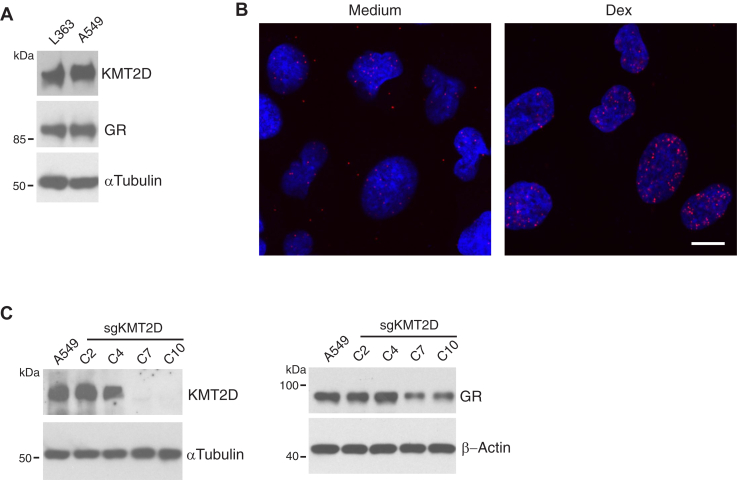
**KMT2D interacts with GR and regulates GR expression in non-lymphoid cells**. *A*, A549 lung epithelial cells were analyzed for KMT2D and GR expression using Western blot. *B*, A549 cells cultured in chamber slides were treated with 100 nM Dex at 37 °C for 30 min. The cells were stained with anti-KMT2D and anti-GR or each single antibody, and PLA spots were imaged by confocal fluorescence microscopy. The images are representative of 2 independent experiments. Scale bar, 20 μm. *C*, *KMT2D* was targeted in A549 cells using CRISPR/Cas9 system. Subclones were analyzed for KMT2D and GR expression by immunoblotting. The data are representative of 2 independent experiments.

Because KMT2D mutations have been largely associated with hematological malignancies, our initial studies focused on lymphoma and multiple myeloma cells. To ask if KMT2D also regulates GR expression in non-lymphoid malignancies, we examined A549 lung adenocarcinoma cells, which are known to be GC-sensitive (33). A549 cells expressed both GR and KMT2D protein at levels comparable to L363 cells (Fig. 7*A*). PLA detected some KMT2D/GR interactions, which markedly increased within the nucleus after Dex treatment (Fig. 7*B*). As in the other cells tested, CRISPR/Cas9 targeting of *KMT2D* resulted in a decrease in GR expression (Fig. 7*C*). We conclude that KMT2D stabilization of GR expression is not limited to neoplastic cells in the hematopoietic lineage.

## Discussion

Glucocorticoid-induced thymocyte death was one of the earliest recognized forms of apoptosis (34). Despite many years of study, the molecular mechanisms underlying GC-induced apoptosis are not completely understood, and indeed other types of regulated cell death, including necroptosis, autophagy, and ferroptosis, have also been reported to contribute to GC-induced cell death (8, 35, 36, 37). What is indisputable, however, is that engagement of the GR initiates a cascade of events leading to cell death, which explains why *NR3C1* sgRNAs were the top hits on the CRISPR screen. With the assistance of co-activators or co-repressors, ligand-activated GR promotes or suppresses the transcription of a myriad of genes. Cell death is likely the outcome of the coordinated action of multiple pathways and proteins that regulate cell survival. In fact, GR-mediated transcriptional enhancement and repression both contribute to GC-induced cell death (8). In sensitive cells, GCs primarily engage the intrinsic mitochondrial apoptosis pathway, in which BCL-2 family proteins are the central intermediaries (8). Several studies suggested that GR-mediated upregulation of *BIM* transcription plays a role in GC-induced apoptosis (11, 12, 13). However, sgRNAs that target pro-apoptotic BCL2 family BH-3-only genes (*BIM*, *BID*, *PUMA*, and *BIK*) or apoptosis effectors (*BAX* and *BAK* and caspases) (38) were not highly enriched in the CRISPR screen. The complexity and redundancies among these genes and pathways may explain why none of these individual genes stood out. Consistent with this, the gene we identified and validated as "pro-apoptotic", *KMT2D*, encodes a protein that acts on the initial and non-redundant molecule in the pathway, the GR itself.

The GR is constitutively expressed in a wide range of tissues and its activity is precisely regulated to deliver accurate control of gene transcription (39). One important mechanism is *via* post-translational modifications, including phosphorylation, ubiquitination, sumoylation, and acetylation, which control GR localization, protein stability, and DNA binding (39, 40). In particular, the GR is known to undergo ubiquitination-dependent proteasomal degradation under both steady-state conditions and in response to GC stimulation (28, 29, 30), which serves to restrain its transcriptional effects (28, 30). The ubiquitin-conjugating enzyme UbcH7, and the ubiquitin-protein ligases CHIP (41), E6-AP (42), and mdm2 (43) have been reported to be responsible for ubiquitinating the GR under these conditions. We found that KMT2D increased GR half-life, at least in part by inhibiting its proteasomal degradation. Although KMT2D is primarily localized to the nucleus, cell fractionation experiments detected a cytoplasmic component as well. Co-immunoprecipitation of KMT2D and the GR has previously been reported in human pigment epithelial cells, although it was not enhanced by exposure to Dex (44). In contrast, we found that in hematopoietic cells KMT2D interacted with the GR at low levels at the nuclear periphery and/or cytoplasm, which is where the GR resides in the absence of GCs, and that nuclear co-localization was enhanced by exposure to GCs. As cells are constantly exposed to glucocorticoid levels that fluctuate in response to diurnal rhythms and stressors, there should be ample opportunity for KMT2D and the GR to interact. KMT2D is an enormous protein (5537 amino acids with a predicted molecular mass of about 600 kDa), and its interaction with the GR may physically prevent access of the ubiquitination machinery (*e.g.* the E2 UbcH7 and the E3 CHIP, E6-AP and mdm2).

A previous study found that KMT2D interacted with the constitutively nuclear estrogen receptor (ER). This interaction was almost totally dependent on the presence of estrogen. In that case, KMT2D regulated gene transcription by acting as a coactivator for ligand-activated ER transcriptional activity and modulated ER-dependent cell growth (24). Several histone acetyl transferases (HATs) such as GRIPS, Tip60, and p300 interact with the GR and act as transcriptional co-activators (45). It was reported that p300 interacts with the compass-like protein complexes that include KMT2C/2D and brings them to enhancer regions to regulate gene transcription (46). KMT2D has also been shown to interact with the transcriptional regulator p53 (47, 48). Nonetheless, we demonstrated here that the chromatin-modifying enzymatic activity of KMT2D was not required and, therefore KMT2D did not regulate GR functions *via* an epigenetic mechanism, but rather by post-translationally upregulating its expression.

Induction of apoptosis and inhibition of cell proliferation represent the main activities of synthetic GCs such as Dex and prednisolone in the treatment of a wide range of pathological conditions. GCs are used in combination with chemotherapeutic agents as the frontline treatment for lymphoid malignancies and other types of cancer. However, their efficacy is often compromised by GC resistance. GC resistance is often caused by gene mutations or polymorphisms and can be acquired during treatment (8). Many mechanisms have been proposed for GC resistance, including decreased GR levels, reduced affinity for GCs, altered post-translational modifications, impaired translocation, altered transcriptional activity and coregulator expression, imbalance between pro- and anti-apoptotic proteins, signaling pathway interaction changes, epigenetic changes, and/or metabolism dysregulation (8, 49). Although reduced GR levels are thought to be an important cause of GC resistance (8, 9), *NR3C1* mutations are not frequently detected in GC-resistant patients (8, 9, 50, 51). Our study demonstrated that knocking out *KMT2D* downregulated endogenous GR levels and conferred resistance to GC-induced apoptosis in multiple myeloma cells. Our data also showed that lower GR levels correlated with natural gene mutation-mediated KMT2D protein loss in cells from DLBCL patients. PI3K pathway activation has been linked with GC resistance. In one study (52), it was reported that AKT phosphorylates GR, which hinders its translocation from cytosol to the nucleus in response to GC stimulation and confers T-ALL cells with GC resistance. In another (53), reduced GC sensitivity was attributed to a decrease in GR levels mediated by activated PI3Ks. It has also been reported that AKT interacted with and phosphorylated KMT2D, which downregulated KMT2D activity in ER-dependent gene transcription in breast cancer (54). Based on the observations here, it is an interesting possibility that PI3K may regulate GC sensitivity *via* its action on KMT2D.

Heterozygous KMT2D mutations cause Kabuki syndrome, a genetic disorder that is sometimes accompanied by autoimmune dysfunction (55, 56). The Kabuki syndrome-related pathogenic KMT2D variants are distributed across nearly the entire protein, indicating that the mechanisms behind Kabuki syndrome go well beyond defects in its methyltransferase activity. The heterogeneity of KMT2D variants, combined with the heterogenic clinical manifestations that make Kabuki syndrome difficult to diagnose, also suggest that KMT2D plays a much broader cellular role than its traditional function in chromatin modification (55). KMT2D is also one of the most frequently mutated genes across a number of neoplasms, including breast cancer, non-Hodgkin's lymphoma (NHL) such as DLBCL and follicular lymphoma, bladder cancer, prostate cancer, colorectal cancer, esophageal squamous cell cancer, T cell lymphoma, and acute myeloid lymphoma (55, 57, 58, 59). KMT2D mutations are associated with poor prognosis in patients with DLBCL (60, 61), mantle cell lymphoma (62), and T cell lymphoblastic lymphoma (63). A portion of lymphoma-associated KMT2D mutations result in truncated proteins that lack the C-terminal SET domain and thus methyltransferase activity. However, some lymphoma-associated variants are missense mutations that produce one or two amino acid changes outside of the SET domain. These amino acid changes may be physically far away from the C-terminus, where almost all the known functional regions are concentrated and unlikely to affect methyltransferase activity (60, 64, 65). Interestingly, a comprehensive analysis of gene mutation landscapes in multiple myeloma cell lines revealed that KMT2D missense mutations far away from the SET region are associated with resistance to GC-induced cell death but increased sensitivity to lenalidomide, another immunomodulatory drug used to treat myelomas and lymphomas (64). Epigenetic regulation of chromatin accessibility has also been linked with GC resistance (66, 67). However, a comprehensive study of epigenetic landscapes in acute lymphoblastic leukemia (ALL) found no role for KMT2D mutations in GC resistance-associated chromatin accessibility changes (66). These observations are consistent with our finding that methyltransferase activity is not responsible for KMT2D regulation of GR levels and GC sensitivity.

It is interesting to note that although the CRISPR screen was designed to discover pro-apoptotic genes downstream of the GR, the top hit was *NR3C1* itself, and the *KMT2D* gene was not identified because of apoptotic activity but because of its role in maintaining GR protein levels. Among the enriched genes was the tumor suppressor, WT1, whose mutations are also associated with leukemia (68). However, specifically targeting *WT1* with a sgRNA transduction did not affect cell survival in the presence of Dex. Among the untested candidates is *SUMO3*, which is intriguing because SUMOylation regulates GR specificity by modulating the chromatin accessibility of GR-binding sites (69, 70) or inhibits GR transcriptional activity through recruiting co-repressors (71). Another enriched gene, *COPRS*, encodes a protein that is required for the methyltransferase activity of protein arginine methyltransferase (PRMT) 5 to catalyze arginine methylation of histones and other substrates (72). A recent study revealed that GR is methylated by PRMT5 in cells (73). The functional significance of GR methylation by PRMT5 has not been determined, but PRMT1 methylation of the ER is required for ER interaction with PI3K and downstream AKT activation (74). Further studies will have to determine if these genes or others that were identified as targets in the CRISPR screen participate in GC-induced cell death.

## Experimental procedures

### Antibodies

Rabbit anti-KMT2D (ABE1867), mouse monoclonal anti-Flag (M2), anti-β-actin, anti-α-tubulin, and anti-lamin A/C were purchased from MilliporeSigma. Mouse monoclonal anti-GR (G5) was purchased from Santa Cruz Biotechnology. Rabbit anti-GR and HRP conjugated anti-ubiquitin was purchased from Cell Signaling Technology.

### Cell lines

Human Jurkat (T cell leukemia) cells, L363 and MM1S cells (multiple myelomas, gifts from Beverly Mock, NCI), and A549 cells (lung carcinoma, ATCC) were cultured in RPMI 1640 (GIBCO) supplemented with 10% heat-inactivated fetal bovine serum (FBS), 2 mM L-glutamine, 50 μM 2-mercaptoethanol, and 100 μg/ml gentamicin (complete medium). 293T cells (ATCC) were cultured in a complete medium containing Dulbecco's Modified Eagle Medium (DMEM, GIBCO). SUDHL2 (ATCC), SUDHL5, SUDHL6, and SUDHL10 DLBCL cells (gifts from Mark Raffeld, NCI) were cultured in a complete medium containing Iscove's Modified Dulbecco's Medium (IMDM).

### Single guide (sg) RNA cloning

Single guide RNAs were cloned into the LentiGuide-Puro (Addgene #52963) and LentiCRISPRv2-neo (Addgene #98292) plasmids as described (75). Briefly, the plasmids were digested with FastDigest Esp3I, and the sgRNA oligonucleotides and their reverse-complement oligonucleotide were annealed, ligated with the Esp3I-digested plasmid, and transformed into Stbl3 cells (ThermoFisher Scientific). The resulting plasmids were purified and sequenced to confirm the proper sgRNA sequence was inserted downstream of the U6 promoter in the CRISPR vectors.

### Pooled CRISPR screening

Stable Jurkat^GR-Cas9^ cells were established by sequentially transducing Jurkat^WT^ cells with a GR-expressing retroviral pMRX-IRESThy1.1 vector (76) and then a Cas9-expressing lentiviral vector. Lentivirus of the Brunello pooled sgRNA library (77) was produced by the NCI LASP Genome Modification Core. Virus titers were measured by infection of cells with serially diluted virus. Jurkat^GR-Cas9^ cells were infected with lentivirus of Brunello sgRNA library (78) in the presence of 4 μg/ml polybrene. Multiplicity of infection (MOI) was set to 0.3 to 0.4 for transduction of a single sgRNA per cell. To maintain the representation of sgRNAs during screening, the number of cells was 1000 times more than the sgRNA number in the library. The sgRNA library-transduced cells were treated with 1 μg/ml puromycin for 5 days and then cultured with 0.1 μM Dex for 3 days. The cells that survived Dex treatment were replated and expanded in the presence of 0.05 μM Dex. Genomic DNA was extracted from triplicates of 10^7^ Dex-treated and 4 × 10^7^ untreated sgRNA library-transduced cells using the DNeasy blood and tissue kit (QIAGEN). sgRNA sequencing was performed by the Genomics Technology Laboratory, Fredrick National Laboratory for Cancer Research. Briefly, PCR amplification of sgRNAs was done using P5-ARGON and P7-KERMIT primers, and Titanium Taq DNA Polymerase (639242, Takara Bio), and Qubit HS DNA kit (ThermoFisher Scientific) was used to quantitate the library concentration. The libraries were sequenced on an Illumina NextSeq200 with 100 +8 cycles. The sequence reads were analyzed with Perl scripts for known and unknown sgRNA inserts.

### Site-directed mutagenesis of plasmid DNA

The size of a plasmid containing full-length *KMT2D* cDNA is too large to efficiently perform PCR-based mutagenesis. Therefore, fragmented *KMT2D* cDNAs were used for mutagenesis. The cDNA fragment encoding the KMT2D C-terminus was purified after digesting pCMV6Entry-*KMT2D* with Xba I and Not I restriction enzymes and cloned into pcDNA3. The EcoR I-digested cDNA fragment encoding the KMT2D N-terminus was cloned into pCMVTag2B. For the re-expression of *KMT2D* in Cas9-edited cells, the sgRNA targeting sequence and protospacer adjacent motif (PAM) sequence in *KMT2D* were mutated to prevent Cas9 targeting while preserving protein amino acid sequence. DNA mutagenesis was carried out using a Q5 site-directed mutagenesis kit per the manufacturer’s instructions (New England Biolabs). Primers for mutagenesis reactions were designed using the manufacturer’s online tool, NEBaseChanger. The sequence-confirmed KMT2D cDNA mutants were assembled back into the original KMT2D expression vector at Xba I and Not I sites for the C-terminal fragment and at the EcoR I site for the N-terminal fragment by subcloning.

### Flow cytometry and flow-based cell sorting

Five million Jurkat cells transduced with the pMRX-IRESThy1.1 retroviral plasmid containing human *NR3C1* open-reading frame were stained with PE-anti-Thy1.1 antibody for 30 min on ice and sorted for Thy1.1-positive cells with a FACSAria (BD Bioscience). Sorted cells were subsequently cloned by limiting dilution. For experiments using intracellular staining to analyze GR expression, approximately 10^6^ cells were centrifuged, and the cells were resuspended in 100 μl of ice-cold Cytofix/Cytoperm (BD Biosciences). The cells were stained by incubating with Alexa647-conjugated anti-GR antibody overnight at 4 °C. Cells were then washed and resuspended in FACS buffer. For detection of apoptosis, cells were stained with FITC-Annexin V and propidium iodide (PI) using FITC-Annexin V/Dead Cell Apoptosis Kit (ThermoFisher Scientific) following the manufacturer’s instructions. Data were acquired using LSR Fortessa SORP flow cytometers with FACSDiva software (BD Biosciences) and analyzed with FlowJo software (BD Bioscience).

### Cell transfection, transduction, and derivation of stable cell lines

293T cells were transfected using Lipofectamine 2000 (ThermoFisher Scientific) following the manufacturer’s instructions. pCMV6Entry-KMT2D plasmid in which the cDNA sequence targeted by the KMT2D sgRNA was mutated was transfected into KMT2D-deficient Jurkat^GR^ cells *via* electroporation at 260 V for 20 s using Gene Pulser Xcell (Bio-Rad Laboratories). For virus production, retroviral or lentiviral plasmids were transfected into 293T cells along with packaging plasmids using Lipofectamine 2000. Retrovirus- or lentivirus-containing supernatants were collected and used to infect Jurkat or L363 cells. To create stable cell lines, cells that were transduced or transfected were selected with 1 μg/ml puromycin or 500 μg/ml geneticin for 10 to 14 days. Stable transductants or transfectants were cloned by limiting dilution and analyzed for protein expression by immunoblotting or flow cytometry.

### Gene knockout using CRISPR-Cas9

Jurkat, L363, and A549 cells were edited using CRISPR–CAS9 engineering technology to generate KMT2D knockout cell lines. Briefly, Jurkat^GR-Cas9^ cells were transduced with lentiGuide-puro with guide RNA against *KMT2D* or other genes. After puromycin selection, cells were cloned by limiting dilution. L363 and A549 cells were transduced with LentiCRISPRv2-neo with *KMT2D* sgRNA and cloned after geneticin selection. The cells were screened *via* immunoblotting to confirm the absence of the target proteins.

### Quantitative RT-PCR

Total RNA was prepared using an RNeasy kit (Qiagen) and quantified by NanoDrop. Reverse transcription was performed using SuperScript IV reverse transcriptase (ThermoFisher Scientific). Gene expression was quantified in quadruplicate using PowerUp SYBR Green (Applied Biosystems). The following primers were used: GR, Fwd, 5′-CAGCAGTGAAATGGGCAAAG-3′; Rev, 5′-AGGAGCAAAACACAGCAGGT-3′; *GAPDH*, Fwd, 5′- ACAACTTTGGTATCGTGGAAGGAC-3’; Rev, 5′-GGGATGATGTTCTGGAGAGC C -3′. Quantification reactions were done on a QuantStudio 6 QPCR thermocycler (ThermoFisher Scientific) and relative expression determined using the ΔΔCt method. *GAPDH* was used for normalization.

### Immunoblotting, immunoprecipitation, and co-immunoprecipitation

Cells were lysed with RIPA lysis buffer (25 mM Tris-HCl, pH 7.4, 1% Triton X-100, 0.5% Na-deoxycholate, 0.1% SDS, 150 mM NaCl, 1 mM EDTA, 20 mM NaF, 1 mM sodium orthovanadate) supplemented with protease inhibitor cocktail (Roche Diagnostics) and normalized for protein content using a Bradford protein assay (Bio-Rad) (79). To determine GR ubiquitination, RIPA cell lysates were normalized for protein concentration, SDS was added to 1% concentration, and the lysates were heated at 95 °C for 5 min to disrupt non-covalent protein interactions. The lysates were then diluted to an SDS concentration of 0.1% and immunoprecipitated with anti-GR (G5) by incubation at 4 °C for 4 h, followed by incubation at 4 °C for 2 h with protein G beads. GR/KMT2D protein interactions were assessed *via* co-immunoprecipitation of proteins from RIPA lysates as described (79). For immunoblotting, proteins in immunoprecipitates or cell lysates were resolved by SDS-PAGE and transferred onto nitrocellulose or PVDF membranes that were subsequently incubated with the indicated primary antibodies overnight at 4 °C or 2 h at room temperature. Proteins of interest were visualized using horseradish peroxidase-conjugated anti-rabbit or anti-mouse Ig secondary antibodies (GE Healthcare) and enhanced chemiluminescence (ThermoFisher Scientific). For KMT2D detection, 3 to 8% Tris-acetate NuPAGE gel (ThermoFisher Scientific) was used for protein separation.

### Subcellular fractionation

Cytoplasmic and nuclear fractions were prepared using Minute Cytoplasmic & Nuclear Exaction Kits for Cells (Invent Biotechnologies) according to the manufacturer’s instructions.

### Proximity ligation assay (PLA)

PLA was carried out as described (32), with minor modifications. Briefly, MM1S and A549 cells were plated onto 8-well chamber slides to allow cells to adhere and stimulated with or without 10^-7^ M Dex for 30 min. The cells were fixed with 2% PFA, permeabilized with 0.2% Triton X-100, and stained with rabbit anti-KMT2D, mouse anti-GR, or both at 4 °C overnight. After washing, the secondary PLA probes anti-mouse PLA-minus and anti-rabbit PLA-plus from Duolink In Situ PLA kits (MilliporeSigma) were added for 1 h at 37 °C. Ligation and amplification were performed using Duolink In Situ Orange starter kit (MilliporeSigma). Samples were washed and mounted with ProLong gold antifade mounting medium with DAPI (ThermoFisher Scientific). A Zeiss laser-scanning confocal microscope (LSM 780) was used to visualize the PLA spots. Image analysis and quantitation of cell nuclei and PLA spots were done using Imaris software (Oxford Instruments). Nuclear boundaries and PLA spots were defined by the software based on signal intensity and size.

### Statistics

Analyses were performed using GraphPad Prism (version 10.1.1). Statistical significance was determined with 1-way ANOVA, and data are presented as means with error bars indicating SD.

## Data availability

All data in this study are provided within the manuscript or the Supporting information.

## Supporting information

This article contains supporting information.

## Conflict of interest

The authors declare that they have no conflicts of interest with the contents of this article.
