# Supplementary material for: The histone methyltransferase KMT2D maintains cellular glucocorticoid responsiveness by shielding the glucocorticoid receptor from degradation
Source: J Biol Chem. 2024 Jul 25;300(8):107581. doi: 10.1016/j.jbc.2024.107581 (PMC11350265; doi:10.1016/j.jbc.2024.107581)
Supplement: Supplemental Figs [file mmc1.pdf]

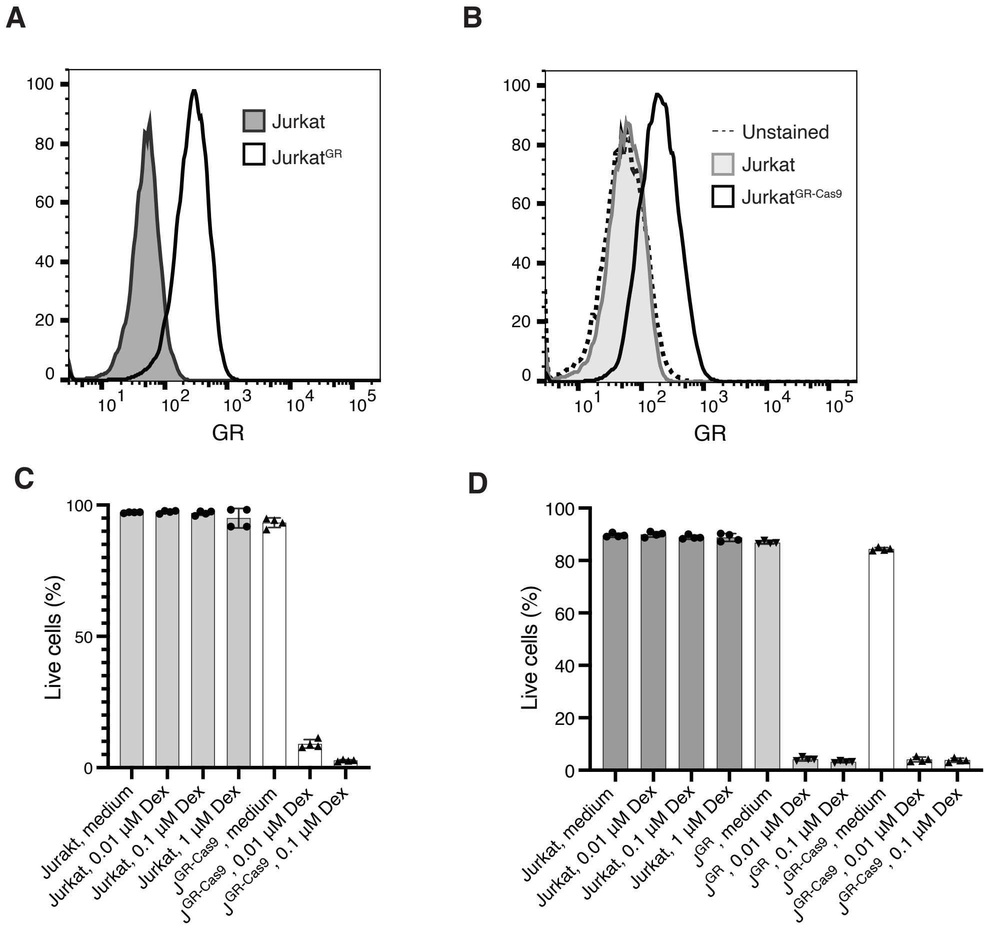

Supplemental Figure 1

Supplemental Figure 1. GR-expressing Jurkat cells are sensitive to GC-induced apoptosis. Jurkat cells were transduced with a GR-expressing retroviral vector alone or along with a Cas9-expressing lentiviral vector. Stable GR-expressing (Jurkat^{GR}) and GR- and Cas9- double expressing Jurkat (Jurkat$^{GR-Cas9}$) clones were established. (A) Jurkat^{GR} and (B) Jurkat$^{GR-Cas9}$ cells were analyzed by flow cytometry for GR expression after intracellular staining with an anti-GR antibody. (C) WT Jurkat cells and Jurkat $^{GR-Cas9}$, and (D) WT Jurkat cells, Jurkat^{GR}, and Jurkat $^{GR-Cas9}$ cells were treated with a series of doses of Dex for 48 h. Live and dead cells were counted after the cells were stained with trypan blue (C) or determined by flow cytometry after the cells were stained with PI and Annexin V-FITC (D). Live (trypan-blue-negative or Annexin V and PI-negative) cell percentages (mean ± SD, n=4) are shown.

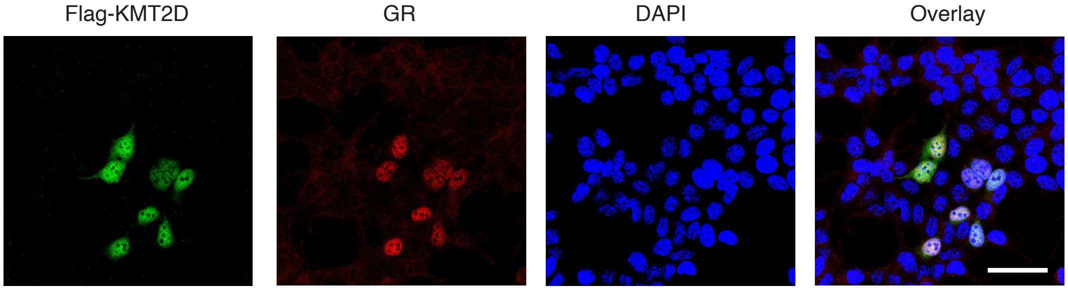

Supplemental Figure 2

Supplemental Figure 2. 293T cells were co-transfected with Flag-tagged KMT2D expression plasmid and GR expression plasmid. The transfected cells are fixed with PFA at 72 h after transfection, stained with mouse anti-Flag Ab and rabbit anti-GR Ab, and imaged by confocal microscopy. Scale bar, 20 μm.

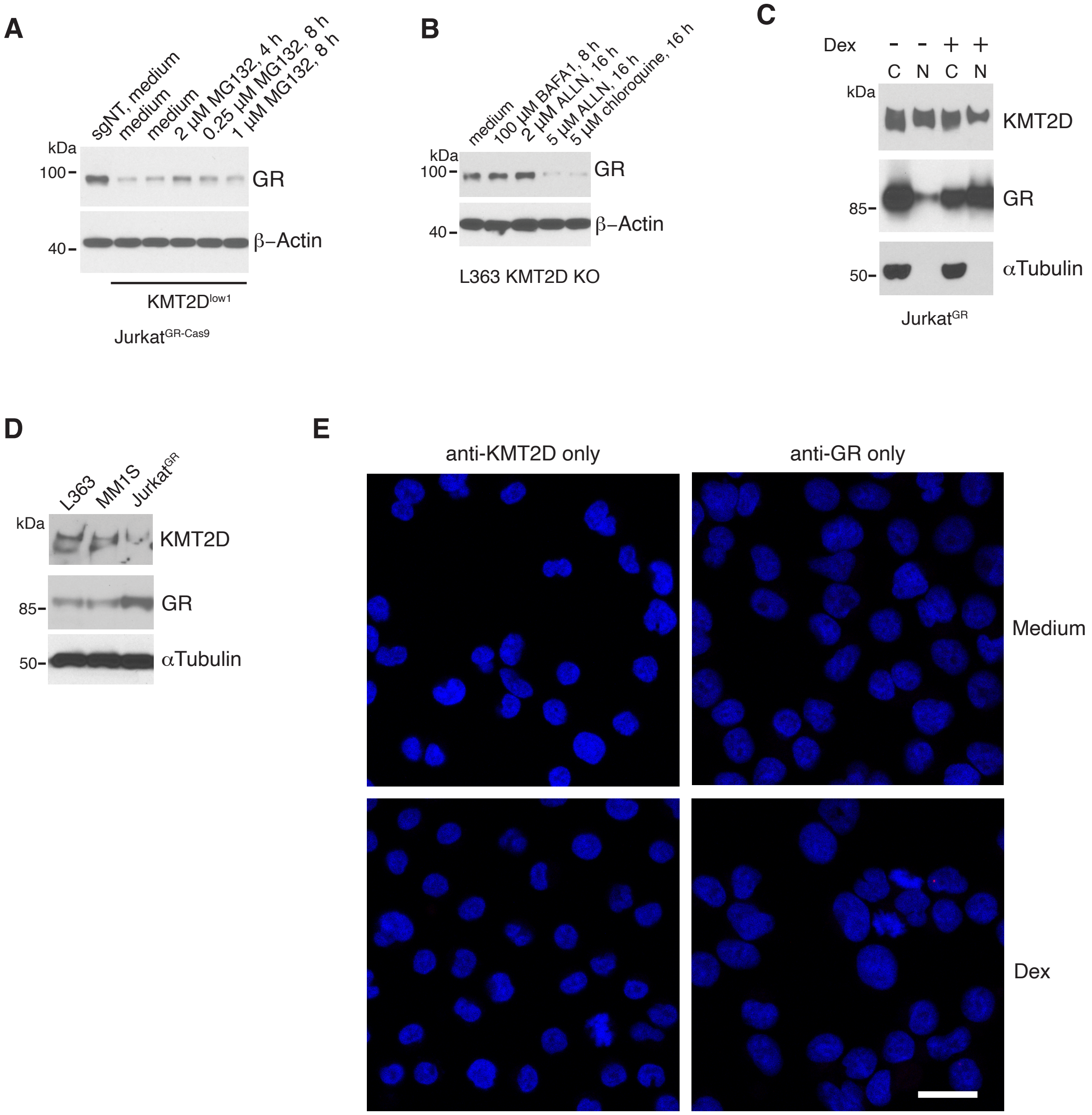

Supplemental Figure 3

Supplemental Figure 3. (A) Control (sgNT-transduced) and KMT2D^{low1} Jurkat^{GR} cells were treated with MG-132 at the indicated doses and durations. Normalized RIPA cell lysates were analyzed for GR protein levels by immunoblotting. (B) KMT2D knockout L363 cells were treated with bafilomycin A (BAFA1), ALLN, or chloroquine. Immunoblotting was performed to determine GR protein levels. (C) Jurkat^{GR} cells were treated with or without 0.1 µM Dex for 30 min, fractionated to separate cytosolic and nuclear lysates, and analyzed for protein localization using immunoblotting as in Fig. 6C. (D) L363, MM1S, and Jurkat^{GR} cells were analyzed for KMT2D and GR expression by immunoblotting. (E) Supplemental to Fig. 6E, Representative pictures of single antibody staining controls for the PLA assay are shown. Scale bar, 10 µm.

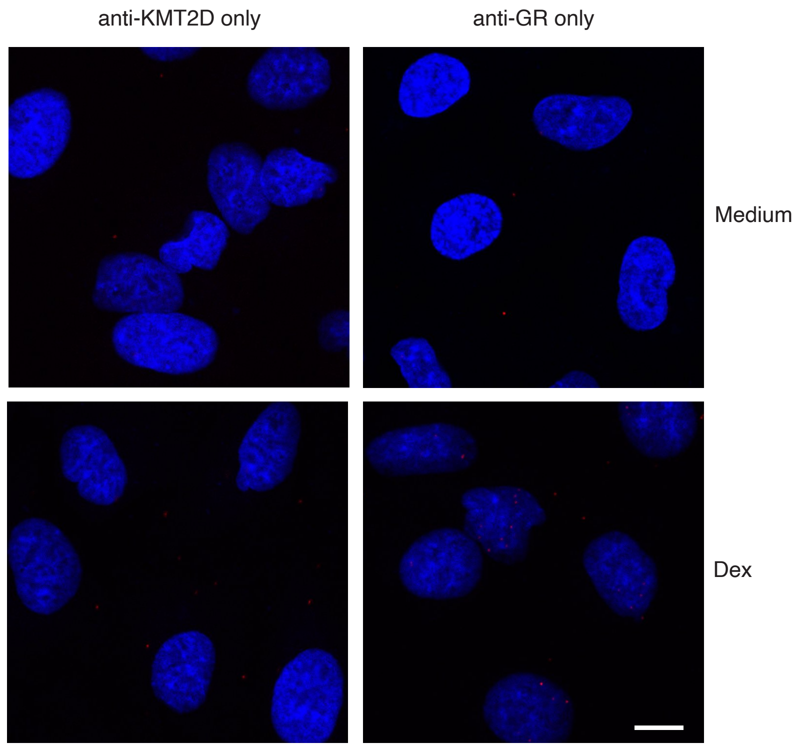

Supplemental Figure 4

Supplemental Figure 4 (related to Fig. 7B). Representative pictures of single antibody staining controls for the PLA assay in Fig. 7B are shown. Scale bar, 20 μm.
